# Efficient Preparation of Small-Sized Transition Metal Dichalcogenide Nanosheets by Polymer-Assisted Ball Milling

**DOI:** 10.3390/molecules27227810

**Published:** 2022-11-12

**Authors:** Qi Zhang, Fengjiao Xu, Pei Lu, Di Zhu, Lihui Yuwen, Lianhui Wang

**Affiliations:** State Key Laboratory of Organic Electronics and Information Displays & Jiangsu Key Laboratory for Biosensors, Institute of Advanced Materials (IAM), Nanjing University of Posts and Telecommunications, 9 Wenyuan Road, Nanjing 210023, China

**Keywords:** high yield, polymer-assisted, ball milling, TMDC nanosheets

## Abstract

Two-dimensional (2D) transition metal dichalcogenide nanosheets (TMDC NSs) have attracted growing interest due to their unique structure and properties. Although various methods have been developed to prepare TMDC NSs, there is still a great need for a novel strategy combining simplicity, generality, and high efficiency. In this study, we developed a novel polymer-assisted ball milling method for the efficient preparation of TMDC NSs with small sizes. The use of polymers can enhance the interaction of milling balls and TMDC materials, facilitate the exfoliation process, and prevent the exfoliated nanosheets from aggregating. The WSe_2_ NSs prepared by carboxymethyl cellulose sodium (CMC)-assisted ball milling have small lateral sizes (8~40 nm) with a high yield (~60%). The influence of the experimental conditions (polymer, milling time, and rotation speed) on the size and yield of the nanosheets was studied. Moreover, the present approach is also effective in producing other TMDC NSs, such as MoS_2_, WS_2_, and MoSe_2_. This study demonstrates that polymer-assisted ball milling is a simple, general, and effective method for the preparation of small-sized TMDC NSs.

## 1. Introduction

2D TMDCs have a unique layered structure in which the layers interact with each other by weak van der Waals force rather than by strong chemical bonding, which makes them different from other materials [1,2,3,4]. 2D TMDCs usually have the general formula of MX_2_, in which transition metal (M) atoms and chalcogen (X) atoms form an X-M-X ‘sandwich’ structure, such as MoS_2_, MoSe_2_, WS_2_, WSe_2_, TiS_2_, or TiSe_2_ [1,5]. Since the interaction between TMDC layers is weak, few-layer or single-layer TMDC NSs can be obtained by physical or chemical exfoliation. These dimension changes in 2D TMDCs cause significant variations in their electronic structures and have a great impact on their physical and chemical properties [2,3]. For example, ultrathin MoS_2_ NSs have high carrier mobility, enhanced photoluminescence, and high catalytic activity [6,7,8,9]. The size and aggregation of TMDC nanosheets also strongly influence the lubrication property in solid lubricants [10,11,12]. Moreover, when the lateral size of TMDC NSs is significantly reduced, typically at less than 20 nm, their properties are influenced by quantum confinement effects [13,14], which make small TMDC NSs promising nanomaterials for applications in optoelectronics, catalysis, energy storage, and biomedicine [3,5,15].

Over the last few decades, various methods have been developed to control the dimensions of TMDC NSs [2,4,16]. Bottom-up methods, such as chemical vapor deposition (CVD) and colloidal synthesis, can be used to synthesize TMDC NSs with various compositions and structures, for which high temperatures and rigorous reaction conditions are usually needed [17,18,19]. As well as bottom-up routes, top-down methods that involve exfoliating bulk TMDCs have also attracted great attention. Novoselov et al. developed a micromechanical exfoliation method for TMDC NSs by using Scotch tape [20]. Although high-quality TMDC NSs can be prepared by using this method, low yield and tedious work limit its use. Chemical exfoliation by alkaline metal intercalation is an efficient top-down approach to prepare ultrathin TMDC NSs [21,22,23]. However, the intercalation reactions usually need to be carried out in strict oxygen-free and water-free conditions, and the as-prepared TMDC NSs often have altered crystal structures with the formation of abundant defects. Ultrasonication-assisted liquid phase exfoliation (LPE) is another popular method that has been extensively explored for the preparation of TMDC NSs due to its simplicity and generality [24,25]. However, the efficiency of LPE is relatively low, especially for the preparation of TMDC NSs with small sizes [24,25,26]. Ball milling is another top-down method to produce TMDC NSs with the potential for large-scale production [27,28,29,30]. Compared with the normal force-dominated LPE method, ball milling utilizes both shear force and normal force provided by the milling balls to exfoliate and pulverize the layered TMDCs [31,32]. Similar to the LPE method, the low yield of nanosheets is also a common issue due to the limited milling ball–material contact interface and the reaggregation of nanosheets during ball milling. Although sonication-assisted exfoliation has been used together with ball milling, the improvement of the yield is still limited and the operation becomes complicated [28,33,34]. Therefore, a novel method for highly efficient preparation of small TMDC NSs with high yield and simplicity is still needed.

**Scheme 1:**
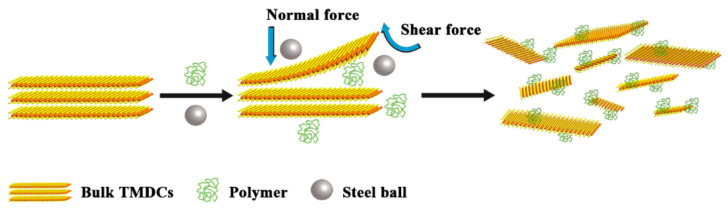
Preparation of small TMDC NSs by polymer-assisted ball milling.

In this report, we develop a polymer-assisted ball milling strategy for the efficient preparation of TMDC NSs with small sizes (Scheme 1). By using carboxymethyl cellulose sodium (CMC) as a solid intermedium in the ball milling process, WSe_2_ NSs with different average sizes from about 8 nm to 40 nm were successfully prepared with a total yield of over 60%. The influence of the polymer, rotation speed, and milling time on the size and yield of WSe_2_ NSs was studied. This method provides polymer surface modification during preparation, which gives the as-prepared WSe_2_ NSs good colloidal stability. Moreover, this method can also be used to prepare other TMDC NSs, such as MoS_2_, MoSe_2_, and WS_2_. Our study demonstrates a novel method for the preparation of small-sized TMDC NSs with high yields by using polymer-assisted ball milling, which is efficient, simple, general, and scalable. 

## 2. Results and Discussion

### 2.1. WSe_2_ NSs Prepared by CMC-Assisted Ball Milling

As illustrated in Scheme 1, polymers are mixed with bulk TMDC materials in a steel jar to assist the dry ball milling process. The shear force provided by the steel balls leads to the exfoliation of the layered materials, while the normal force fragments the nanosheets. During ball milling, the polymers act as an intermedium to transfer the impact from steel balls to TMDC materials and enhance the interaction between steel balls and TMDC materials. Moreover, the polymer molecules can adsorb on the surface of TMDC NSs and reduce their reaggregation, which also improves the exfoliation efficiency. Polymer-assisted ball milling not only decreases the thickness and lateral size of layered TMDCs but also produces homogeneous TMDC–polymer composites. Without further ultrasonication treatment, the as-prepared TMDC NSs can easily disperse in water and form stable colloidal dispersions. TMDC NS aqueous dispersions were first centrifuged at low speed to remove the large aggregates, and the supernatant was further centrifuged at different speeds to obtain TMDC NSs with different sizes.

**Figure 1:**
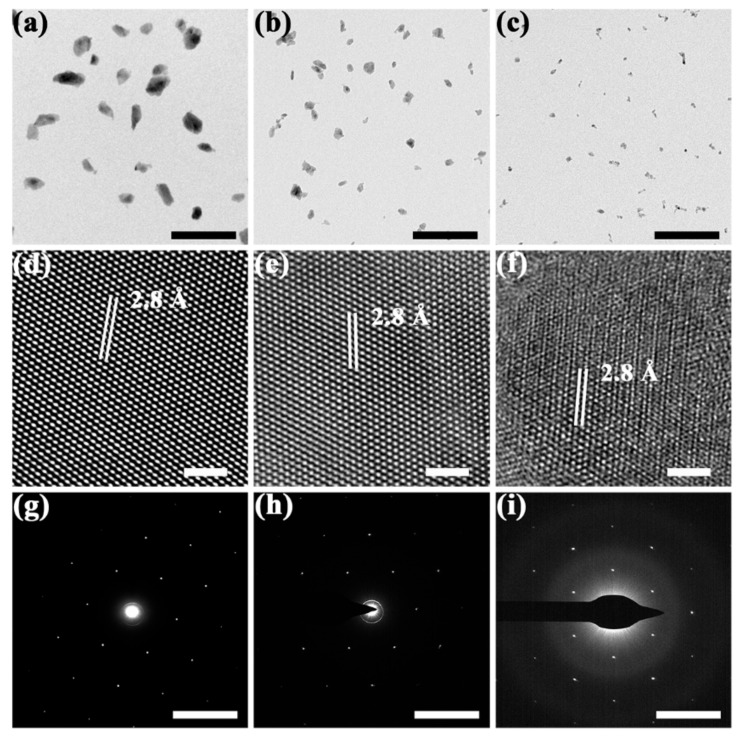
(**a**–**c**) TEM, (**d**–**f**) HRTEM, and (**g**–**i**) SAED images of WSe_2_ NSs with different sizes prepared by CMC–assisted ball milling at different centrifugation speeds: (**a**,**d**,**g**) WSe_2_–Low (10,000 rpm for 1 h), (**b**,**e**,**h**) WSe_2_–Medium (16,000 rpm for 1.5 h), (**c**,**f**,**i**) WSe_2_–High (21,000 rpm for 4 h). Scale bars: 200 nm for (**a**–**c**), 2 nm for (**d**–**f**), and 5 1/nm for (**g**–**i**).

CMC was first explored to prepare WSe_2_ NSs by ball milling. As a carboxymethyl functionalized cellulose, CMC is an abundant, cheap, and environmentally friendly material and has been extensively used in various industrial applications [35]. After ball milling, CMC-WSe_2_ NSs composites can easily form aqueous dispersions due to the electrostatic repulsion force provided by CMC. After gradient centrifugation at different rotation speeds, a series of small WSe_2_ NSs were obtained and named as WSe_2_-Low (10,000 rpm), WSe_2_-Medium (16,000 rpm), and WSe_2_-High (21,000 rpm), respectively. TEM was used to investigate the morphology of WSe_2_ NSs. Figure 1a–c and Figure S1 show that WSe_2_ NSs have uniform sheet-like morphology with average sizes of 39.66 ± 13.63 nm (WSe_2_-Low), 20.02 ± 6.28 nm (WSe_2_-Medium), and 7.90 ± 3.11 nm (WSe_2_-High), respectively. As shown by the HRTEM images in Figure 1d–f, WSe_2_ NSs have a clear crystalline structure and the lattice spacing of 2.8 Å can be assigned to the (100) plane of 2H-WSe_2_ [36]. The six-fold SAED patterns (Figure 1g–i) indicate that the WSe_2_ NSs with different sizes have the same hexagonal symmetry structure, suggesting no significant change in the structure during the ball milling process. 

**Figure 2:**
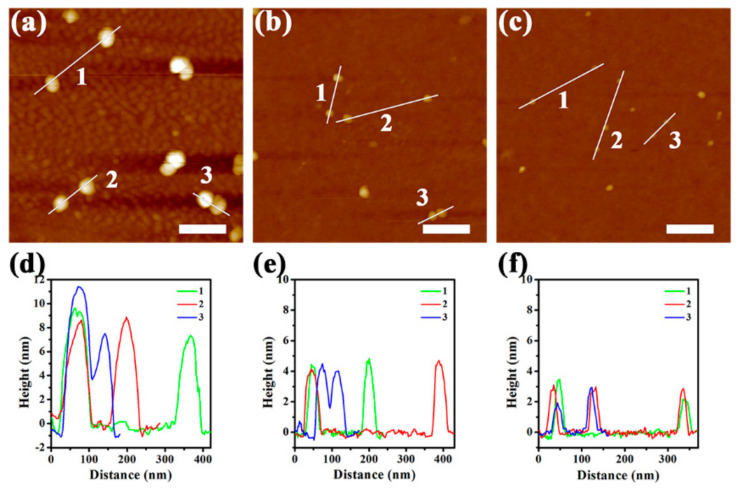
(**a**–**c**) AFM images and (**d**–**f**) corresponding height profiles of WSe_2_ NSs with different sizes prepared by CMC–assisted ball milling after gradient centrifugation: (**a**,**d**) Wse_2_–Low, (**b**,**e**) Wse_2_–Medium, (**c**,**f**) Wse_2_–High. Scale bar: 200 nm.

Atomic force microscopy (AFM) was used to investigate the thickness of WSe_2_ NSs. AFM images (Figure 2 and Figure S2) show that the average thickness of WSe_2_ NSs gradually decreases from 8–10 nm (WSe_2_-Low) to 2–3 nm (WSe_2_-High) according to the increase in centrifugation speed from 10,000 rpm to 21,000 rpm. Hence, small-sized WSe_2_ NSs with different thicknesses can be obtained by varying the rotation speed during gradient centrifugation. Since CMC molecules can still absorb on the surface of WSe_2_ NSs after purification, the apparent thickness of WSe_2_ NSs detected by AFM may be a little higher than the exact true values. 

**Figure 3:**
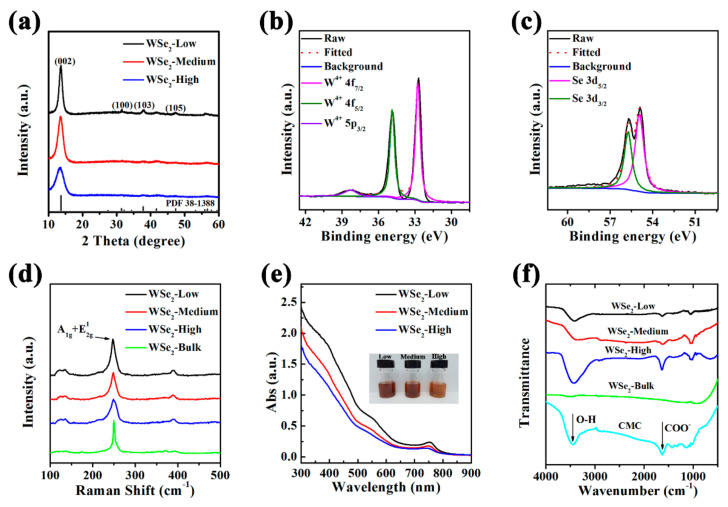
(**a**) XRD patterns of WSe_2_ NSs with different sizes. XPS spectra of WSe_2_ NSs (WSe_2_–Low) for (**b**) W 4f and W 5p, and (**c**) Se 3d. (**d**) Raman spectra of bulk WSe_2_ and WSe_2_ NSs with different sizes. (**e**) UV–vis–NIR absorption spectra and photos of WSe_2_ NSs aqueous dispersions (inset). (**f**) FT–IR spectra of WSe_2_ NSs, bulk WSe_2_, and CMC.

The structure, composition, and properties of the WSe_2_ NSs were further studied. As shown by the XRD patterns in Figure 3a, diffraction peaks at 13.6°, 31.4°, 37.8°, and 47.4° belong to the (002), (100), (103), and (105) planes according to the standard diffraction data of WSe_2_ (JCPDS, 38-1388). The broadening and weakening of these diffraction lines originate from the size and thickness reduction of WSe_2_ NSs after ball milling [37,38]. XPS was used to investigate the chemical state of the as-prepared WSe_2_ NSs. As shown by the core-level XPS spectra of W in Figure 3b, the doublet peaks near 33 eV and 35 eV belong to W^4+^ 4f_7/2_ and W^4+^ 4f_5/2_ of 2H-WSe_2_, while the binding energy peak located at about 38 eV can be ascribed to W^4+^ 5p_3/2_ [39]. The absence of doublet peaks for W^6+^ between 36 and 38 eV indicates that no WOx formed during ball milling [40]. As illustrated in Figure 3c, the doublet peaks near 55 eV and 56 eV belong to Se 3d_5/2_ and Se 3d_3/2_ of 2H-WSe_2_ [41]. Similarly, WSe_2_ NSs with other sizes (WSe_2_-Low and WSe_2_-High) have the same chemical states of W and Se as WSe_2_-Medium (Supplementary Materials Figure S3), which implies that WSe_2_ NSs were prepared by polymer-assisted ball milling mainly through physical exfoliation and fragmentation rather than the mechanochemical way. Raman spectra of WSe_2_ NSs (Figure 3d) show that the characteristic peak located at about 250 cm^−1^ can be ascribed to the degenerate A_1g_ (out-of-plane) and E^1^_2g_ (in-plane) vibrational modes, suggesting the few-layer structure of WSe_2_ NSs [39,41]. UV-vis-NIR spectra of WSe_2_ NSs (Figure 3e) indicate that the absorption peaks near 750 nm blueshift with the size and thickness reduction, similar to previous reports [42,43]. As the FT-IR spectra of WSe_2_ NSs depict in Figure 3d, IR absorption bands at 3430 cm^−1^ and 1630 cm^−1^ can be assigned to the stretching vibration of the hydroxyl group (−OH) and asymmetric vibration of the carboxyl group (COO−) of CMC [44,45], respectively, which suggests the existence of CMC molecules on the surface of WSe_2_ NSs. Due to the hydrophilicity and electrostatic repulsion of CMC, WSe_2_ NSs are stable in phosphate buffer saline (PBS) and cell culture medium (DMEM) (Supplementary Materials Figure S4), and no obvious precipitation can be observed even after being stored for months in water, which suggests their good colloidal stability in biological environments.

### 2.2. The Influence of Experimental Conditions on Polymer-Assisted Ball Milling

The influence of the experimental conditions of ball milling on the size and yield of WSe_2_ NSs was studied. Due to the important role of the polymer during ball milling, different polymers were first studied. As shown in Supplementary Materials Figure S5, WSe_2_ NSs prepared by using different polymers all have uniform morphology and good dispersity with an average size smaller than 100 nm. Table 1 indicates that the total yield of WSe_2_ NSs by CMC-assisted ball milling is 62.68%, while the yields of WSe_2_ NSs using F127, PVP, and PEG are 12.34%, 32.08%, and 11.89%, respectively. Moreover, the lateral size of WSe_2_ NSs using CMC is much smaller than those using other polymers. The superiority of CMC for the ball milling process may originate from its unique structure. Previous studies have revealed that the hydroxyl and carboxyl groups of polymers, such as alginate, bovine serum albumin, and glycan, have a strong interaction with the surface of TMDC NSs, and the synergy of the repetitive units of the polymers also enhances this interaction [46,47,48]. Compared with F127, PVP, and PEG, CMC has much more hydroxyl and carboxyl groups, which may provide a stronger interaction between the surface of WSe_2_ NSs and polymers and significantly improve the efficiency of ball milling. Milling time and rotation speed during ball milling also play important roles in the morphology and yield of the WSe_2_ NSs. As shown in Table 1 and Supplementary Materials Figure S6, the lateral size of WSe_2_ NSs generally reduces with increased milling time, while total yields of WSe_2_ NSs are nearly the same (~60%), suggesting that the milling time mainly influences the size of WSe_2_ NSs rather than the yield. As shown in Supplementary Materials Figure S7 and Table 1, the size of most WSe_2_ NSs decreases from about 60 nm to 30 nm when the rotation speed of the ball mill increases from 400 rpm to 800 rpm. Meanwhile, the total yield of WSe_2_ NSs increases from about 30% to 60% once the rotation speed has exceeded 650 rpm. Therefore, both the size and yield of WSe_2_ NSs can be easily adjusted by varying the rotation speed and milling time during ball milling, suggesting the controllability of this method.

### 2.3. The Preparation of other TMDC NSs by Polymer-Assisted Ball Milling

**Figure 4:**
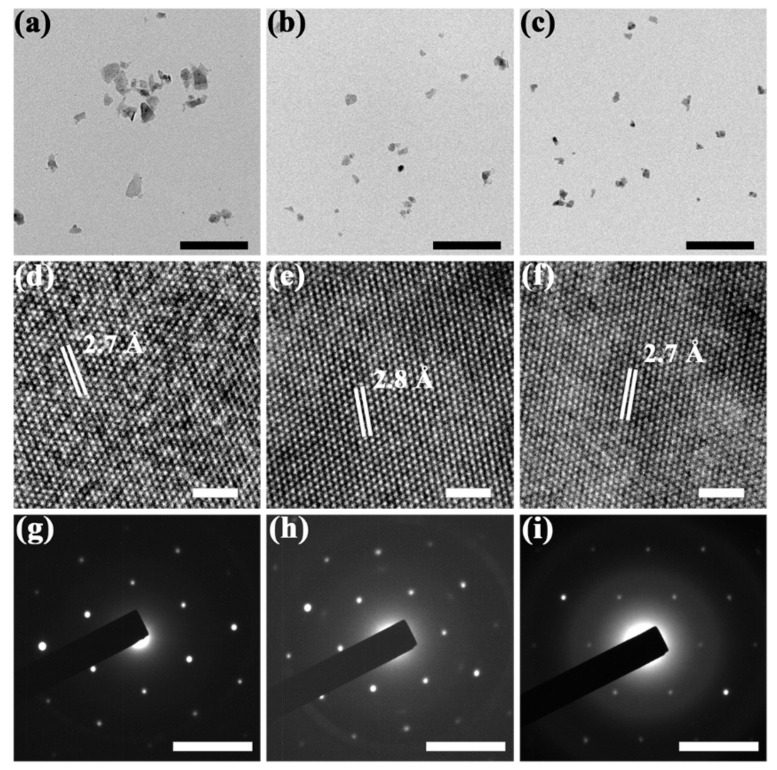
(**a**–**c**) TEM, (**d**–**f**) HRTEM, and (**g**–**i**) SAED images of MoS_2_, MoSe_2_, and WS_2_ NSs prepared by CMC–assisted ball milling: MoS_2_ (**a**,**d**,**g**), MoSe_2_ (**b**,**e**,**h**), and WS_2_ (**e**,**f**,**i**). Scale bars: 200 nm for (**a**–**c**), 2 nm for (**d**–**f**), and 5 1/nm for (**g**–**i**).

**Figure 5:**
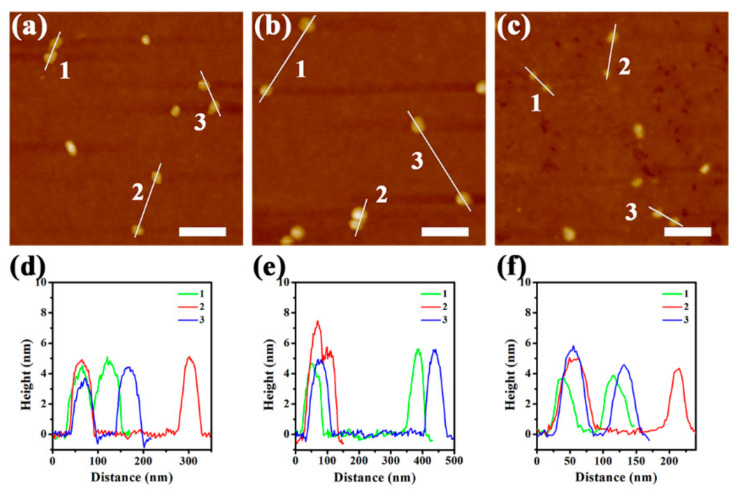
AFM images and corresponding height profiles of MoS_2_, MoSe_2_, and WS_2_ NSs prepared by CMC–assisted ball milling: (**a**,**d**) MoS_2_, (**b**,**e**) MoSe_2_, (**c**,**f**) WS_2_. Scale bar: 200 nm.

The feasibility of polymer-assisted ball milling for the preparation of other TMDC NSs was investigated. As shown in Figure 4a–c, the MoS_2_, MoSe_2_, and WS_2_ NSs prepared by CMC-assisted ball milling have uniform morphology and good dispersity. These TMDC NSs obtained by medium centrifugation speed (16,000 rpm) have lateral sizes similar to WSe_2_-Medium (~20 nm) (Figure S8, Supplementary Materials), suggesting the general applicability of this method. HRTEM images (Figure 4d–f) show that MoS_2_, MoSe_2_, and WS_2_ NSs all have a clear crystalline structure with identical lattice spacings to previous reports [36,46]. The SAED patterns (Figure 4g–i) further indicate the hexagonal symmetry structure of these TMDC NSs, which is characteristic of the crystal structure of the 2H phase [24,36]. As shown in Figure 5 and Supplementary Materials Figure S9, the average thickness of these TMDC NSs is in the range of 4–6 nm, suggesting they have a similar few-layer structure to WSe_2_ NSs.

**Figure 6:**
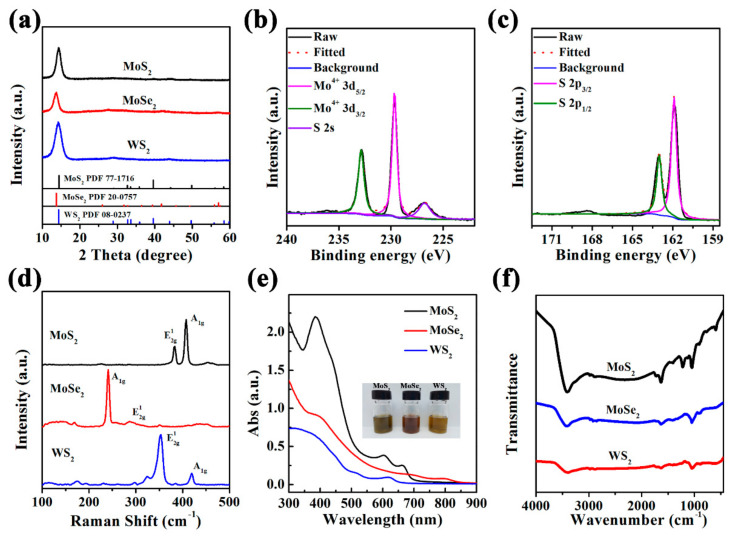
(**a**) XRD patterns of MoS_2_, MoSe_2_, and WS_2_ NSs prepared by CMC–assisted ball milling. XPS spectra of MoS_2_ NSs for Mo 3d (**b**) and S 2p (**c**) core level energy regions. (**d**) Raman spectra of MoS_2_, MoSe_2_, and WS_2_ NSs. (**e**) UV–vis–NIR absorption spectra with the photos (inset) and (**f**) FT–IR spectra of MoS_2_, MoSe_2_, and WS_2_ NSs.

To further confirm the successful preparation of the MoS_2_, MoSe_2_, and WS_2_ NSs, structure, composition, and property characterizations were performed. As illustrated in Figure 6a, diffraction peaks at 14.3° (MoS_2_), 13.7° (MoSe_2_), and 14.3° (WS_2_) belong to the (002) plane, respectively, similar to the standard diffraction data. The weakening of (100), (103), and (105) lines may originate from the size and thickness reduction of these TMDC NSs [37]. The doublet peaks in the XPS spectrum of Mo (Figure 6b) near 229 eV and 232 eV correspond to Mo^4+^ 3d_5/2_ and Mo^4+^ 3d_3/2_ of 2H-MoS_2_, respectively; and the weak peak near 227 eV corresponds to S 2s [34,49]. The absence of the peak near 237 eV for Mo^6+^ 3d suggests no oxidation of MoS_2_ [40]. The doublet peaks near 162 eV and 163 eV in Figure 6c can be ascribed to the S 2p_3/2_ and S 2p_1/2_ of 2H-MoS_2_, respectively. In addition, the high-resolution XPS spectra (Supplementary Materials Figure S10) further confirm the successful preparation of 2H phase MoSe_2_ NSs and WS_2_ NSs, while slight oxidation was found for WS_2_ NSs [40]. Raman spectra of MoS_2_, MoSe_2_, and WS_2_ NSs (Figure 6d) reveal the characteristic peaks for MoS_2_ (408 cm^−1^ and 382 cm^−1^), MoSe_2_ (241 cm^−1^ and 287 cm^−1^), and WS_2_ (420 cm^−1^ and 354 cm^−1^), which belong to their A_1g_ and E^1^_2g_ vibrational modes, respectively [33]. Figure 6e shows the UV-vis-NIR spectra of the TMDC NSs aqueous dispersions and their photos under ambient light (inset). The distinct absorption peaks located near 800 nm (MoSe_2_), 632 nm (MoS_2_), and 620 nm (WS_2_) originate from the A exciton transition, similar to the reports of few-layer TMDC NSs [43,50]. Figure 6f and Figure S11 show the FT-IR spectra of the TMDC NSs and their bulk materials. The IR absorption bands near 3410 cm^−1^ and 1630 cm^−1^ can be assigned to the stretching frequency of OH and COO groups from CMC adsorbed on the surface of the TMDC NSs.

## 3. Materials and Methods

### 3.1. Chemicals

WSe_2_ powder (99.8%) was purchased from Alfa Aesar. MoS_2_ powder (99%), MoSe_2_ powder (99.9%), WS_2_ powder (99%), Pluronic F-127, and polyvinyl pyrrolidone (PVP) were obtained from Sigma-Aldrich (St. Louis, MO, USA). Carboxymethyl cellulose sodium (CMC) and polyethylene glycol (PEG) were purchased from Aladdin and Macklin, respectively. Ultrapure water (18.2 MΩ, Billerica, MA, USA) was used to prepare aqueous solutions in this study.

### 3.2. Characterization

Transmission electron microscopy (TEM) images were obtained by using a HT7700 at 120 kV (Hitachi, Tokyo, Japan). High-resolution transmission electron microscopy (HRTEM) and selected area electron diffraction (SAED) images were obtained using field emission electron microscopes (Talos F200x, FEI, 200 kV, Waltham, MA, USA). Atomic force microscopy (AFM) images were acquired on Nanoscope IIIa (Bruker, Billerica, MA, USA). X-ray diffraction (XRD) patterns were recorded on an X-ray diffractometer (D8 Advance A25, Bruker, Billerica, MA, USA) with Cu Kα radiation (λ = 1.54178 Å). X-ray photoelectron spectroscopy (XPS) was performed on KRATOS Axis Supra (Shimadzu, Kyoto, Japan) with Al Kα (hν = 1486.6 eV) as the excitation source. Raman characterization was carried out on a micro-Raman spectroscopy system (inVia, Renishaw, Wotton-under-Edge, UK) equipped with a 532 nm laser. Ultraviolet-visible-near infrared (UV-vis-NIR) absorption spectroscopy was performed on a UV-3600 spectrophotometer (Shimadzu, Kyoto, Japan). Fourier transform infrared (FT-IR) spectra were recorded on a FT-IR spectrometer (Perkin Elmer, Waltham, MA, USA). The concentration of TMDC NSs was determined by using an inductively coupled plasma optical emission spectrometer (ICP-OES, Optima 5300DV, Perkin Elmer, Waltham, MA, USA).

### 3.3. Preparation of WSe_2_ NSs by CMC-Assisted Ball Milling

WSe_2_ powder (0.1 g) and CMC (1.0 g) were mixed and put into the steel jar of a planetary ball mill (Tianchuang, Changsha, China) for ball milling at 650 rpm (35× *g*) for 12 h. Then, 40 mL ultrapure water was added into the jar and mixed. The aqueous suspensions of WSe_2_ were first centrifuged at 3000 rpm (765× *g*) for 30 min to remove the large aggregates. Then, the supernatant was centrifuged at 10,000 rpm (8497× *g*) for 1 h. The sediment was collected and redispersed in water, while the supernatant was further centrifuged at 16,000 rpm (21,752× *g*) for 1.5 h. Similarly, the sediment was collected and the supernatant was then centrifuged at 21,000 rpm (37,471× *g*) for 4 h. Each sediment was further purified more than three times by centrifugation to remove the redundant CMC. Finally, the sediments were dispersed in ultrapure water and stored at 4 °C. WSe_2_ NSs obtained at different centrifugation speeds (low speed at 10,000 rpm (8497× *g*), medium speed at 16,000 rpm (21,752× *g*), and high speed at 21,000 rpm (37,471× *g*)) are referred to as WSe_2_-Low, WSe_2_-Medium, and WSe_2_-High, respectively.

### 3.4. WSe_2_ NSs Prepared by Using Different Polymers during Ball Milling

The preparation of WSe_2_ NSs by using different polymers, including F127, PVP, and PEG, has similar experimental conditions to CMC-assisted ball milling. Since the stabilization abilities of different polymers are different, the centrifugation conditions used to purify WSe_2_ NSs were adjusted according to the polymer used. For F127, WSe_2_ NSs were obtained by gradient centrifugation at 5000 rpm (2124× *g*) for 20 min (WSe_2_-Low), 7500 rpm (4779× *g*) for 20 min (WSe_2_-Medium), and 10,000 rpm (8497× *g*) for 30 min (WSe_2_-High). For PVP, WSe_2_ NSs were obtained by gradient centrifugation at 10,000 rpm (8497× *g*) for 1 h (WSe_2_-Low), 16,000 rpm (21,752× *g*) for 2 h (WSe_2_-Medium), and 21,000 rpm (37471× *g*) for 4 h (WSe_2_-High). For PEG, WSe_2_ NSs were obtained by gradient centrifugation at 5000 rpm (2124× *g*) for 20 min (WSe_2_-Low), 7500 rpm (4779× *g*) for 20 min (WSe_2_-Medium), and 10,000 rpm (8497× *g*) for 30 min (WSe_2_-High).

### 3.5. Preparation of Other TMDC NSs 

MoS_2_, MoSe_2_, and WS_2_ NSs were prepared using similar procedures to the CMC-assisted ball milling of WSe_2_ NSs, except that the centrifugation conditions were adjusted according to the different materials.

## 4. Conclusions

In summary, we have developed a novel polymer-assisted ball milling method for the efficient preparation of TMDC NSs with small sizes. The as-prepared WSe_2_ NSs by using CMC have small sizes (8–40 nm) with high yield (over 60%), which is not easy to achieve by using other top-down methods. The high efficiency of this method is attributed to the enhanced interaction of the 2D TMDCs and the milling balls because of the polymer used during ball milling. The size and thickness of the WSe_2_ NSs can be adjusted by changing the rotation speed and milling time during ball milling and the centrifugation conditions during purification. Moreover, this polymer-assisted ball milling method can also be used to prepare other TMDC NSs, such as MoS_2_, WS_2_, and MoSe_2_. The as-prepared TMDC NSs have good colloidal stability in PBS and cell culture medium. This study provides a highly efficient, simple, general, and scalable method for the preparation of TMDC NSs with small sizes, which may also be used for other 2D materials.

## Figures and Tables

**Table 1:** Average lateral size and yield of WSe_2_ NSs prepared by polymer-assisted ball milling under different experimental conditions.

Polymer	Speed (rpm)	Time (h)	Size (nm)/Yield (%)			Total Yield (%)
			WSe_2_-Low	WSe_2_-Medium	WSe_2_-High	
CMC	650	12	39.66/26.32	20.02/26.18	7.90/10.18	62.68
F127	650	12	93.20/7.68	72.39/3.84	48.73/0.82	12.34
PVP	650	12	46.29/23.48	19.05/8.39	14.87/0.21	32.08
PEG	650	12	47.05/8.81	37.00/2.21	30.76/0.87	11.89
CMC	650	6	55.04/22.34	35.28/25.60	21.54/14.36	62.30
CMC	650	24	24.06/32.52	12.41/23.72	7.68/3.82	60.06
CMC	400	12	61.05/26.92	51.44/1.32	35.78/0.56	28.80
CMC	800	12	28.26/23.61	15.82/21.34	7.46/13.92	58.87

## Data Availability

The raw data will be available from the corresponding authors upon reasonable request.

## Supplementary Materials

The following supporting information can be downloaded at: https://www.mdpi.com/article/10.3390/molecules27227810/s1, Figure S1: Large-scale TEM images of WSe2 NSs prepared by CMC-assisted ball milling at 650 rpm for 12 h and centrifuged under different conditions; Figure S2: Large-scale AFM images of WSe2 NSs prepared by CMC-assisted ball milling at 650 rpm for 12 h and centrifuged under different conditions; Figure S3: XPS spectra of WSe2 NSs prepared by CMC-assisted ball milling at 650 rpm for 12 h; Figure S4: Photographs of CMC-WSe2 NSs dispersed in water, PBS, and DMEM for different times; Figure S5: TEM images of WSe2 NSs prepared by ball milling with different polymers under different centrifugal conditions; Figure S6: TEM images of WSe2 NSs prepared by CMC-assisted ball milling for different times after gradient centrifugation; Figure S7: TEM images of WSe2 NSs prepared by CMC-assisted ball milling with different rotation speeds after gradient centrifugation; Figure S8: Size statistics of (a) MoS2, (b) MoSe2, and (c) WS2 NSs prepared by CMC-assisted ball milling; Figure S9: Large-scale AFM images of (a) MoS2, (b) MoSe2, and (c) WS2 NSs prepared by CMC-assisted ball milling; Figure S10: High-resolution XPS spectra of Mo 3d (a) and Se 3d (b) core level energy regions for MoSe2, and W 4f (c) and S 2p (d) core level energy region for WS2; Figure S11: FT-IR spectra of bulk MoS2, MoSe2, and WS2.

## Sample Availability

Samples of the TMDC NSs are available from the authors.
