# Supplementary material for: Efficient Preparation of Small-Sized Transition Metal Dichalcogenide Nanosheets by Polymer-Assisted Ball Milling
Source: Molecules. 2022 Nov 12;27(22):7810. doi: 10.3390/molecules27227810 (PMC9694012; doi:10.3390/molecules27227810)
Supplement: Supplementary file 1 [file molecules-27-07810-s001.zip › molecules-2037523-supplementary.pdf]

Supporting Information

Efficient Preparation of Small-Sized Transition Metal Dichalcogenide Nanosheets by Polymer-Assisted Ball Milling

Qi Zhang, Fengjiao Xu, Pei Lu, Di Zhu, Lihui Yuwen * and Lianhui Wang *

State Key Laboratory of Organic Electronics and Information Displays & Jiangsu Key Laboratory for Biosensors, Institute of Advanced Materials (IAM), Nanjing University of Posts and Telecommunications, 9 Wenyuan Road, Nanjing 210023, China

* Correspondence: iamlhyuwen@njupt.edu.cn (L.Y.); iamlhwang@njupt.edu.cn (L.W.); Tel.: +86-25-85866333 (L.W.); Fax: +86-25-85866396 (L.W.)

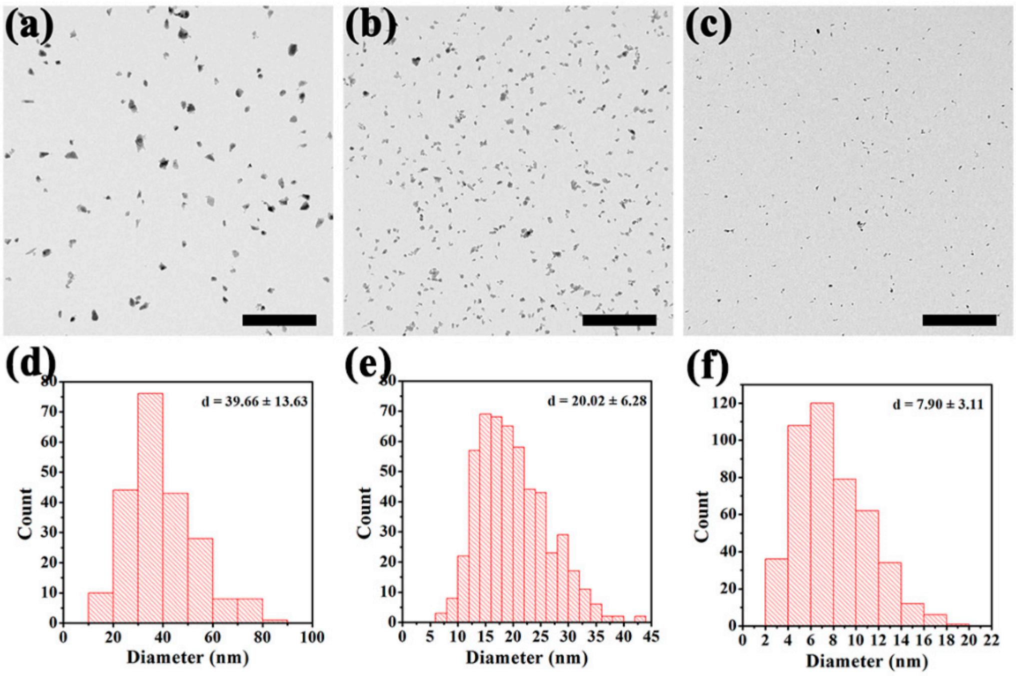

Figure S1 Large-scale TEM images of WSe_2 NSs prepared by CMC-assisted ball-milling at 650 rpm for 12 h and centrifuged at different conditions: (a) 10000 rpm for 1 h, WSe_2-Low; (b) 16000 rpm for 1.5 h, WSe_2-Medium; (c) 21000 rpm for 4 h, WSe_2-High. Scale bar: 500 nm. Size statistics of WSe_2-Low (d), WSe_2-Medium (e), and WSe_2-High (f) based on more than 100 nanosheets.

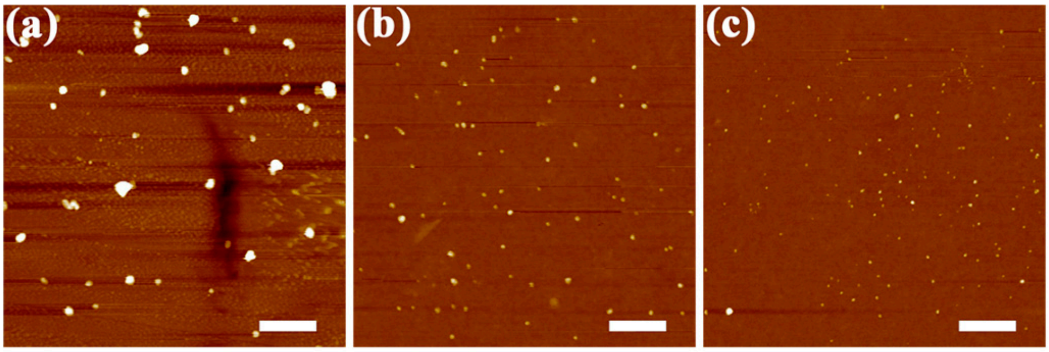

Figure S2 Large-scale AFM images of WSe_2 NSs prepared by CMC-assisted ball-milling at 650 rpm for 12 h and centrifuged at different conditions: (a) WSe_2-Low; (b) WSe_2-Medium; (c) WSe_2-High. Scale bar: 500 nm.

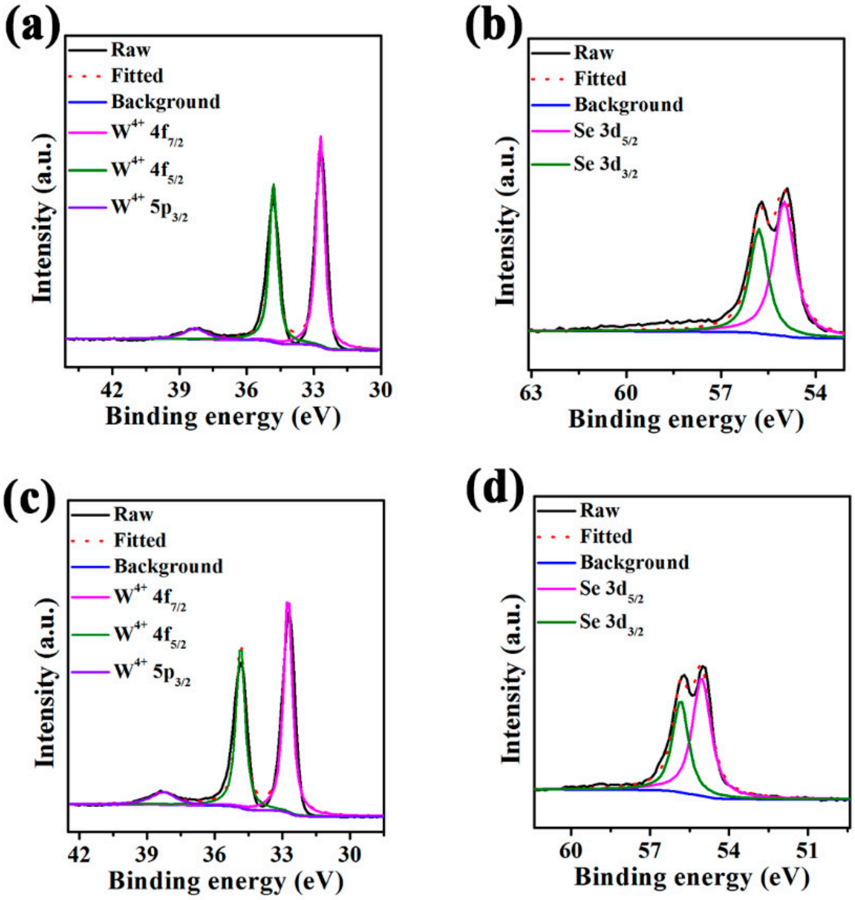

Figure S3 XPS spectra of WSe_2 NSs prepared by CMC-assisted ball-milling at 650 rpm for 12 h: (a–b) WSe_2-Medium, (c–d) WSe_2-High.

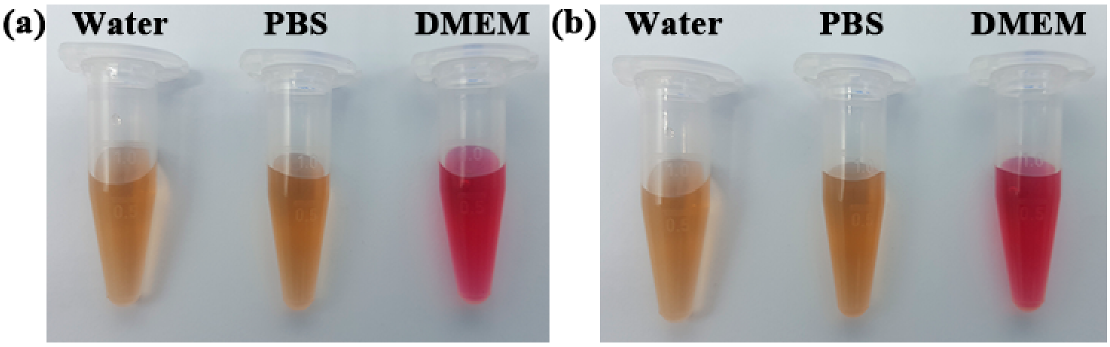

Figure S4 Photographs of CMC-WSe_2 NSs dispersed in water, PBS, and DMEM for different times: (a) 0 h and (b) 24 h.

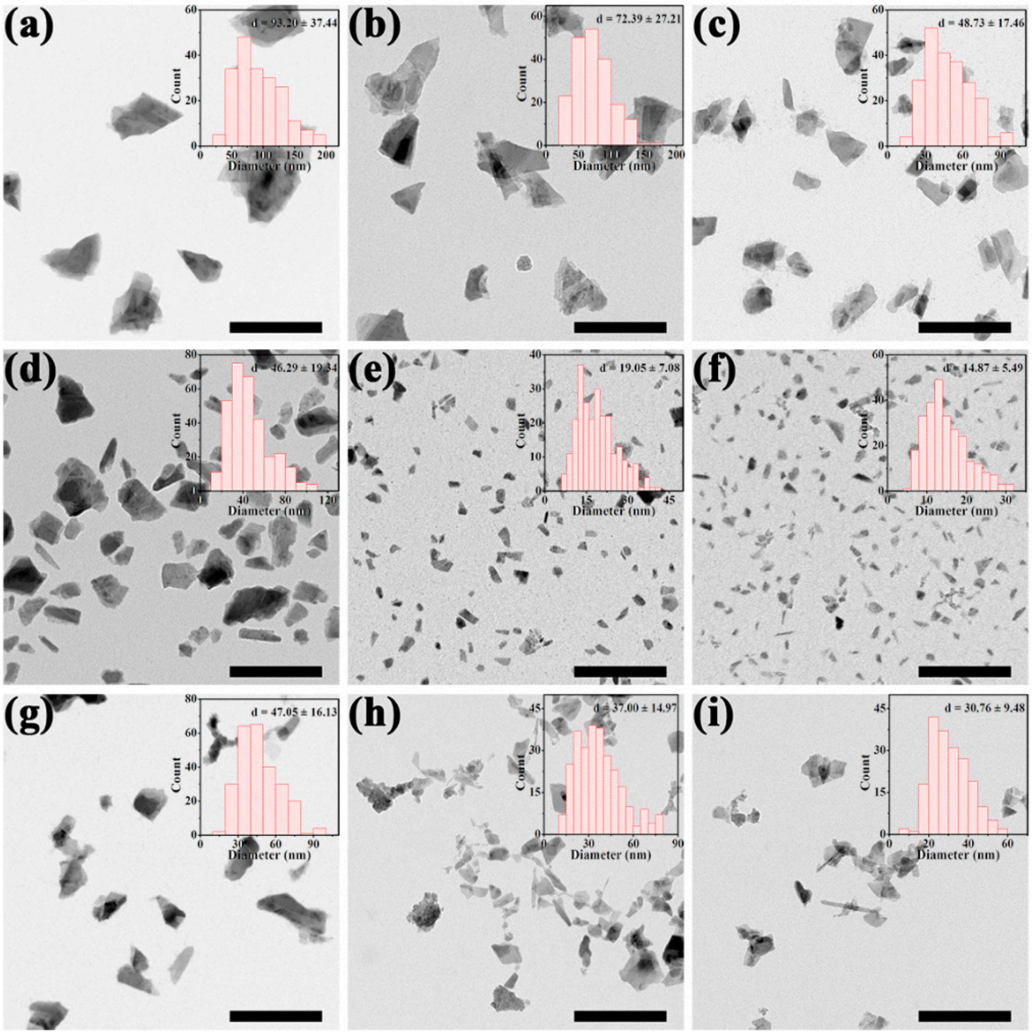

Figure S5 TEM images of WSe_2 NSs prepared by ball-milling with different polymers

at different centrifugal conditions: (a) F127, 5000 rpm for 20 min, WSe_2-Low; (b) F127, 7500 rpm for 20 min, WSe_2-Medium; (c) F127, 10000 rpm for 30 min, WSe_2-High; (d) PVP, 10000 rpm for 1 h, WSe_2-Low; (e) PVP, 16000 rpm for 2 h, WSe_2-Medium; (f) PVP, 21000 rpm for 4 h, WSe_2-High; (g) PEG, 5000 rpm for 20 min, WSe_2-Low; (h) PEG, 7500 rpm for 20 min, WSe_2-Medium; (i) PEG, 10000 rpm for 30 min, WSe_2-High. Scale bar: 200 nm. The insets show the size statistics of WSe_2 NSs based on more than 100 nanosheets from TEM images.

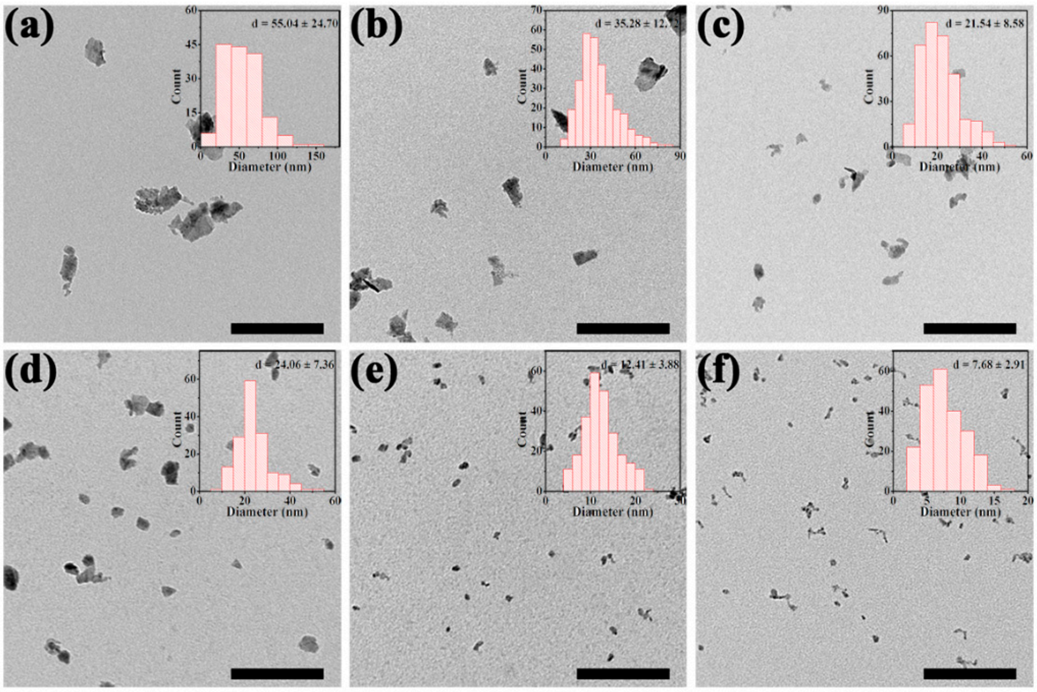

Figure S6 TEM images of WSe_2 NSs prepared by CMC-assisted ball-milling for different times after gradient centrifugation: (a) 6 h, WSe_2-Low; (b) 6 h, WSe_2-Medium; (c) 6 h, WSe_2-High; (d) 24 h, WSe_2-Low; (e) 24 h, WSe_2-Medium; (f) 24 h, WSe_2-High. Scale bar: 200 nm. The insets are size statistics of WSe_2 NSs based on more than 100 nanosheets from TEM images.

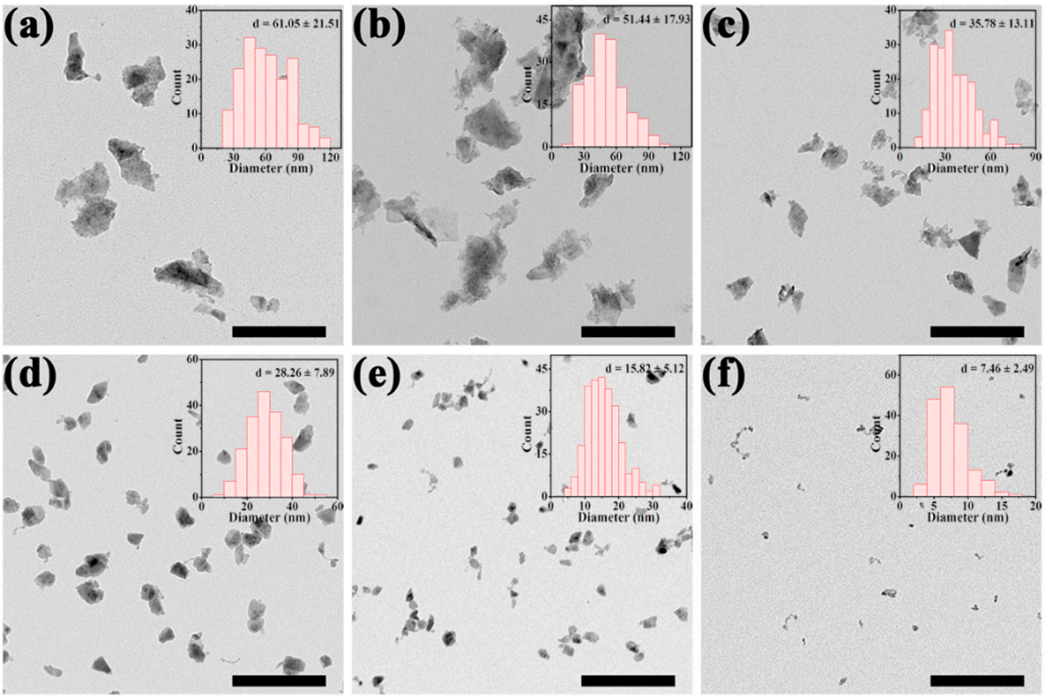

Figure S7 TEM images of WSe_2 NSs prepared by CMC-assisted ball-milling with different rotation speeds after gradient centrifugation: (a) 400 rpm, WSe_2-Low; (b) 400 rpm, WSe_2-Medium; (c) 400 rpm, WSe_2-High; (d) 800 rpm, WSe_2-Low; (e) 800 rpm, WSe_2-Medium; (f) 800 rpm, WSe_2-High. Scale bar: 200 nm. The inset of size statistics is calculated based on more than 100 nanosheets from TEM images.

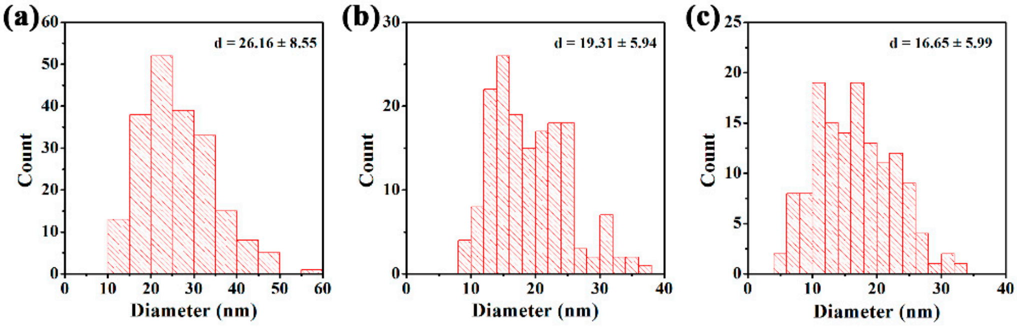

Figure S8 Size statistics of (a) MoS_2, (b) $MoSe_2$, and (c) WS_2 NSs prepared by CMC-assisted ball milling.

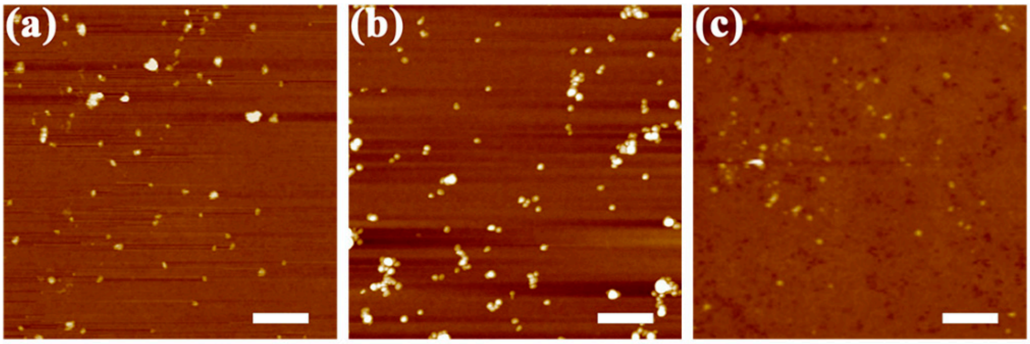

Figure S9 Large-scale AFM images of (a) MoS_2, (b) $MoSe_2$, and (c) WS_2 NSs prepared by CMC-assisted ball-milling. Scale bar: 500 nm.

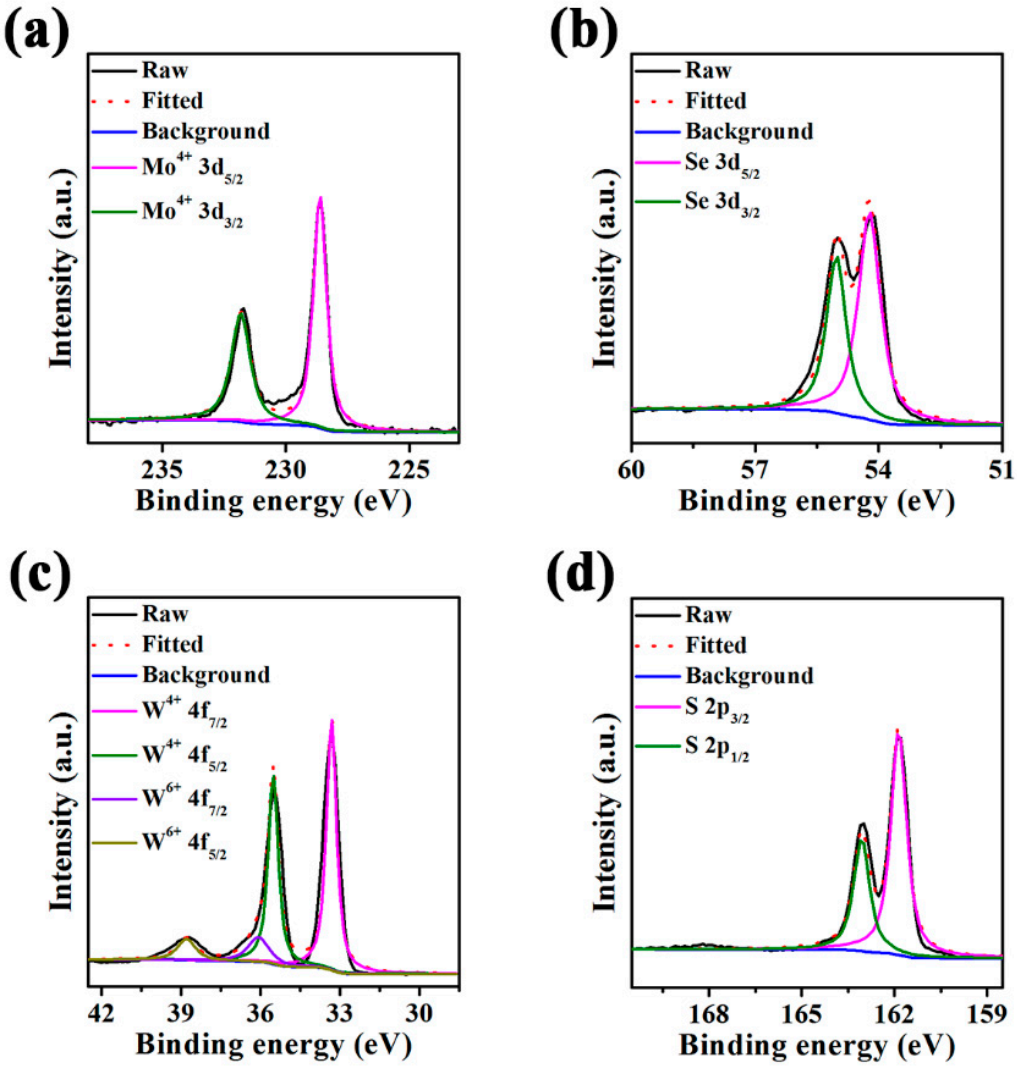

Figure S10 High-resolution XPS spectra of Mo 3d (a) and Se 3d (b) core level energy regions for $MoSe_2$, and W 4f (c) and S 2p (d) core level energy region for WS_2.

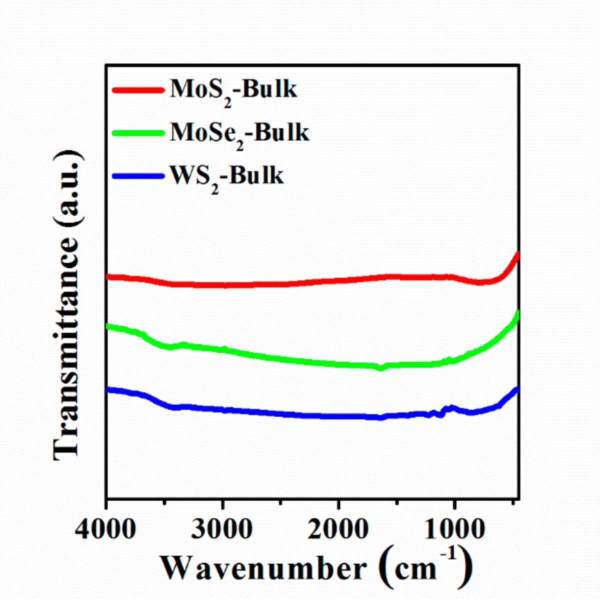

Figure S11 FT-IR spectra of bulk MoS_2, $MoSe_2$, and WS_2.
